# Body mass index decline and cognitive trajectories in mild cognitive impairment: the role of maternal history of Alzheimer’s disease

**DOI:** 10.21203/rs.3.rs-9696646/v1

**Published:** 2026-05-28

**Authors:** Azuquahe Pérez Hernández, Enrique González Dávila, Norberto Rodríguez Espinosa

**Affiliations:** Hospital Universitario Insular de Gran Canaria; University of La Laguna; University of La Laguna

**Keywords:** Alzheimer Disease, Mild Cognitive Impairment, Body Mass Index, Weight Loss, Anthropometric-Metabolic Phenotype, Maternal History, Cognitive Trajectories

## Abstract

**Background:**

Maternal history of Alzheimer’s disease (MH) has been associated with increased risk of Alzheimer’s disease (AD), earlier onset, and distinct neurobiological features. In parallel, an anthropometric-metabolic phenotype characterized by body mass index (BMI) decline and unintentional weight loss has been described as an early feature preceding cognitive decline in the prodromal stages of AD.

**Objective:**

To investigate whether MH is associated with a distinctive anthropometric-metabolic and cognitive phenotype in individuals with mild cognitive impairment (MCI), focusing on longitudinal trajectories of body mass index (BMI), memory, and executive function.

**Methods:**

Participants with MCI from the Alzheimer’s Disease Neuroimaging Initiative (ADNI) were analyzed over up to 24 months of follow-up. Longitudinal changes in BMI, memory, and executive function were examined using repeated-measures ANOVA. MH, APOE4 status, age, sex, and conversion to AD were included as between-subject factors.

**Results:**

Among 397 participants with MCI, 270 (68%) remained clinically stable and 127 (32%) converted to AD during follow-up. APOE4 positivity was more frequent among converters. BMI declined over time, with a steeper reduction observed in individuals who converted to AD and in those with MH. MH was associated with differential longitudinal cognitive trajectories, characterized by subtle non-linear changes in memory performance and relative preservation of executive function. Changes in BMI showed modest associations with memory and executive function trajectories.

**Conclusions:**

MH is associated with distinctive longitudinal patterns of anthropometric-metabolic and cognitive change in individuals with MCI. Incorporating maternal history may help refine phenotypic characterization in prodromal AD, supporting a more nuanced understanding of disease heterogeneity in the prodromal stages.

## Introduction

Alzheimer’s disease (AD) is a multifactorial neurodegenerative disorder shaped by the interaction of genetic, metabolic, and environmental determinants^1^. Family history is one of the strongest non-modifiable risk factors, and converging evidence indicates that maternal history (MH) of AD confers a disproportionately higher risk than paternal history^2,3^. Several studies have demonstrated that cognitively unimpaired individuals with MH show earlier hippocampal atrophy^4,5^, hypometabolism on FDG-PET^6,7^, and greater amyloid burden^8,9^, suggesting a biologically distinct vulnerability profile that may involve mitochondrial or epigenetic mechanisms inherited through the maternal line^2,3^. However, more recent work has yielded mixed results regarding preferential maternal transmission, and this association has been questioned^10,11^.

In parallel, AD is increasingly recognized as a heterogeneous clinical entity with identifiable cognitive subtypes^12^. The most common profile is characterized by predominant impairment in episodic memory with temporomedial involvement, while a subset of patients displays early executive or attentional deficits linked to more widespread or frontally biased patterns of neurodegeneration. Identifying early determinants of these subtypes—particularly in the MCI stage—may improve risk stratification and help clarify mechanistic pathways underlying prodromal AD.

An anthropometric-metabolic phenotype may also play a relevant role in AD vulnerability. Obesity, diabetes, and insulin resistance—conditions with well-documented maternal-line transmission patterns^13,14^—increase AD risk^15,16^. Conversely, unintentional weight loss is frequently observed in prodromal AD and has been reported as a predictor of MCI-to-AD conversion^17–19^. Previous work links weight loss to hippocampal dysfunction^20^, suggesting that BMI decline may reflect early neurodegenerative processes.

Given the intersection among maternal transmission, anthropometric-metabolic vulnerability, and hippocampal involvement, we hypothesized that MCI individuals with maternal history of AD exhibit a distinct clinical phenotype, characterized by: 1) Preferential decline in memory relative to executive functions, consistent with early temporomedial involvement. 2) A characteristic pattern of BMI decline, reflecting underlying anthropometric-metabolic vulnerability.

The objective of the present study was to evaluate the longitudinal evolution of BMI, memory performance, and executive function over 24 months in a well-characterized MCI cohort from ADNI, comparing individuals with and without maternal history. By integrating anthropometric-metabolic and cognitive trajectories, we aimed to determine whether maternal history identifies a specific prodromal AD profile with potential implications for risk prediction and clinical phenotyping.

## Methods

### ADNI Database

This study used data from the Alzheimer’s Disease Neuroimaging Initiative (ADNI), a large, multisite longitudinal project designed to collect detailed clinical, neuropsychological, imaging, and laboratory data from older adults across the cognitive spectrum. ADNI procedures include standardized assessments at regular intervals, encompassing Clinical Dementia Rating (CDR), medical and neurological examinations, neuropsychological batteries, and anthropometric measurements.

We selected all participants diagnosed with mild cognitive impairment (MCI) at baseline. ADNI MCI criteria required Mini-Mental State Examination (MMSE) scores between 24 and 30, subjective memory complaints corroborated by study partners, objective memory impairment on the Logical Memory II subtest adjusted for education, a CDR of 0.5, preserved activities of daily living, and absence of dementia. Full inclusion and exclusion criteria are available on the ADNI website (adni.loni.usc.edu).

### Study Sample and Variables

A total of 400 baseline MCI participants were screened. Three were excluded because of missing BMI data, resulting in a final sample of 397 individuals with available baseline height and weight. Follow-up cognitive and anthropometric data were available for 355 participants at 12 months and 300 at 24 months.

Participants were categorized into two groups based on their cognitive trajectory throughout the study period: 1) stable MCI, defined as individuals who did not convert to AD during the 24-month follow-up period; and 2) converters, defined as individuals who met diagnostic criteria for AD at any follow-up visit. Family history of dementia was extracted from ADNI records when available and categorized as maternal, paternal, both parents, or no family history. For analyses involving maternal influence, we focused on the maternal-only group because paternal and dual-history cases were small (< 7%). BMI (kg/m^2^) was calculated from measured height and weight at each visit. Weight loss was evaluated as the difference between baseline and 24-month weight and BMI. APOE4 genotype: Participants carrying one or two ε4 alleles were classified as APOE4-positive. Age groups followed established ADNI-based categorization: <70 years, 70–80 years, and > 80 years.

### Cognitive Measures

Cognitive performance was assessed using validated ADNI composite indices. Memory was evaluated using the ADNI-Mem composite^21^, derived from the Rey Auditory Verbal Learning Test (RAVLT), ADAS-Cog memory items, MMSE three-word recall, and Wechsler Logical Memory. Executive function was assessed using the ADNI-EF composite^22^, incorporating Trails A and B, digit symbol substitution, digit span backward, category fluency, and clock drawing. Both composites have been validated for sensitivity to longitudinal change in MCI and early AD and show good psychometric properties in ADNI cohorts.

### Statistical Methods

Baseline categorical variables (e.g., sex, family history, APOE4 status) were compared using chi-square tests or Fisher’s exact test when expected cell counts were < 5. Continuous variables (BMI, weight, ADNI-Mem, ADNI-EF, MMSE) were evaluated for normal distribution and compared using t-tests or ANOVA as appropriate. Pairwise comparisons following ANOVA used the Tukey test. Linear relationships between variables were evaluated using Pearson correlation coefficients.

To examine longitudinal trajectories of BMI, ADNI-Mem, and ADNI-EF, we applied repeated-measures ANOVA with time (baseline, 12 months, 24 months) as the within-subject factor; and sex, APOE4 genotype, maternal history, conversion status, and age group as between-subject factors. To further characterize the pattern of change over time, orthogonal polynomial contrasts were utilized to evaluate both linear and quadratic trends. For memory and executive function analyses, BMI change was included as a covariate to assess its influence on cognitive change.

A multivariate analysis of the significant interactions across time was conducted to examine joint evolution of BMI, ADNI-Mem, and ADNI-EF, using the factors above.

All analyses were performed in SPSS 25 for Windows (IBM SPSS, Armonk, NY). Statistical significance was defined as p < 0.05.

## Results

### Participant Characteristics

A total of 397 individuals with MCI were included (mean age 78.6 ± 7.5 years). Of these, 270 (68%) remained clinically stable and 127 (32%) converted to AD over 24 months (Table 1). APOE4 positivity was significantly more frequent in converters (66.1%) compared with stable participants (47.4%; p = 0.001). Memory (ADNI-Mem), executive function (ADNI-EF), and MMSE scores were significantly lower in converters at baseline, 12 months, and 24 months than in stable participants (all p < 0.001; Table 1). Baseline characteristics according to family history are shown in Table 2.

Values are presented as number (%) for categorical variables and mean ± standard deviation for continuous variables. p values refer to comparisons between participants who converted to Alzheimer’s disease and those who remained stable. MCI, mild cognitive impairment; AD, Alzheimer’s disease; BMI, body mass index; MEM (ADNI-Mem), composite memory score; EF (ADNI-EF), composite executive function score; MMSE, Mini-Mental State Examination.

Values are presented as number (%) or mean ± standard deviation. p-values for comparisons across family history groups. BMI: body mass index; MEM (ADNI-Mem): composite of memory function; EF (ADNI-EF): composite of executive function; MMSE: Mini-Mental State Examination.

### Influence of Conversion on BMI and Weight Trajectories

**Figure 1:**
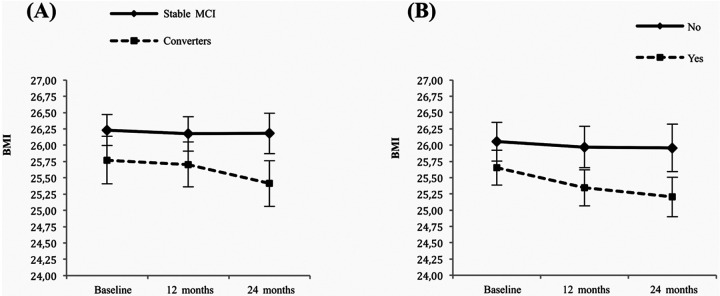
Longitudinal trajectories of body mass index in individuals with mild cognitive impairment. (A) Mean body mass index (BMI) trajectories over 24 months in participants who remained clinically stable (Stable MCI) and those who converted to Alzheimer’s disease during follow-up (Converters). (B) Mean BMI trajectories over 24 months according to maternal history of Alzheimer’s disease (Yes vs No). Values represent estimated means with error bars indicating standard errors.

BMI declined progressively across the cohort over 24 months (time effect, p = 0.019; Table 3, Fig. 1). Converters exhibited a more pronounced reduction in BMI (mean decrease of 0.4 kg/m^2^; from 25.8 ± 4.10 to 25.4 ± 3.71) between baseline and 24 months compared with stable participants, who showed minimal change (26.2 ± 3.92 to 26.2 ± 4.25; Table 1). Weight decline mirrored BMI patterns, with between-group differences approaching statistical significance at 24 months (p = 0.055; Table 1).

Repeated-measures ANOVA revealed a significant time × conversion interaction for BMI (p = 0.022; Table 3), showing that BMI trajectories differed significantly between converters and stable participants (Fig. 1).

Linear and quadratic effects correspond to polynomial contrasts for time. p values < 0.05 were considered statistically significant. Conversion status indicates progression from mild cognitive impairment to Alzheimer’s disease during follow-up. APOE ε4 status reflects carrier vs non-carrier status. Family history refers to maternal history of Alzheimer’s disease.

(A) Mean body mass index (BMI) trajectories over 24 months in participants who remained clinically stable (Stable MCI) and those who converted to Alzheimer’s disease during follow-up (Converters). (B) Mean BMI trajectories over 24 months according to maternal history of Alzheimer’s disease (Yes vs No).

Values represent estimated means with error bars indicating standard errors.

### Maternal History and BMI Decline

Maternal history was associated with BMI evolution. Individuals with maternal history showed a significantly steeper decline over time in BMI (time × maternal history interaction, p = 0.005; Table 3). At 24 months, the maternal-history group had the lowest mean BMI across all family-history categories (25.2 ± 3.82 vs. 26.0 ± 4.08 kg/m^2^ in those without family history; Table 2). These effects remained after accounting for APOE4 status, age, and sex in the repeated-measures model.

Age further modulated BMI trajectories: a significant time × age interaction (p = 0.038) and a three-way interaction between time, maternal history, and age (p = 0.008) indicated steeper BMI decline at older ages in participants with maternal history (Table 3, Fig. 1). A quadratic time × sex interaction for BMI showed a trend toward significance (p = 0.050; Table 3), suggesting sex-specific differences in the rate of weight loss over time.

### Memory Trajectories (ADNI-Mem)

**Figure 2:**
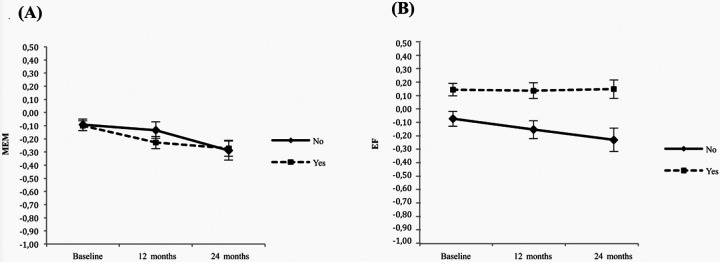
Longitudinal cognitive trajectories according to maternal history of Alzheimer’s disease. (A) Memory performance over 24 months assessed using the ADNI-Mem composite score in participants with and without maternal history of Alzheimer’s disease. (B) Executive function performance over 24 months assessed using the ADNI-EF composite score according to maternal history. Values represent estimated means with error bars indicating standard errors.

Memory performance worsened over time in both groups, with converters showing a markedly steeper decline (time × conversion interaction, p < 0.001; Table 4, Fig. 2A). Baseline ADNI-Mem was significantly lower in converters (− 0.389 ± 0.447) than in stable individuals (0.055 ± 0.589), and the difference widened at 12 and 24 months (all p < 0.001; Table 1).

Across family-history groups, mean ADNI-Mem scores were similar at each time point (Table 2). However, a significant quadratic time × maternal-history interaction was observed for memory (p = 0.036; Table 4), in line with subtle non-linear differences in trajectories between participants with and without maternal history (Fig. 2A).

In models including BMI change (baseline–24 months) as a covariate, the interaction between time and BMI change for ADNI-Mem did not reach statistical significance (p = 0.127; Table 4), although Fig. 2 illustrates lower memory scores in participants with greater BMI loss.

Linear and quadratic effects correspond to polynomial contrasts for time. p values < 0.05 were considered statistically significant. Conversion status indicates progression from mild cognitive impairment to Alzheimer’s disease during follow-up. APOE4 reflects carrier vs non-carrier status. BMI change refers to the difference between baseline and 24-month body mass index.

### Executive Function Trajectories (ADNI-EF)

Executive functioning declined over time in converters (baseline to 24-month change: from − 0.314 ± 0.711 to − 0.803 ± 0.916), while stable individuals showed a small improvement (from 0.083 ± 0.785 to 0.233 ± 0.911; all between-group comparisons p < 0.001; Table 1).

Maternal history was associated with higher mean consistently better executive function scores at all time points. At 24 months, the maternal-history group maintained significantly higher ADNI-EF scores compared with non-maternal-history individuals (− 0.15 ± 1.04 vs. −0.26 ± 1.04; p = 0.032; Table 2).

A significant time × maternal-history interaction was found for ADNI-EF (p = 0.026; Table 4), consistent with modestly divergent EF trajectories between those with and without maternal history (Fig. 2B).

(A) Memory performance over 24 months assessed using the ADNI-Mem composite score in participants with and without maternal history of Alzheimer’s disease. (B) Executive function performance over 24 months assessed using the ADNI-EF composite score according to maternal history.

Values represent estimated means with error bars indicating standard errors.

### Multivariate Models

The multivariate repeated-measures analysis revealed:

BMI trajectories were independently influenced by maternal history (time × maternal history, p = 0.005), conversion status (time × conversion, p = 0.022), APOE4 (time × APOE4, p = 0.028), and age (time × age, p = 0.038), with a quadratic time × sex interaction at the threshold of significance (p = 0.050; Table 3).

Memory trajectories (ADNI-Mem) were influenced by conversion (time × conversion, p < 0.001), with a trend-level interaction with APOE4 (time × APOE4, p = 0.076) and a significant quadratic interaction with maternal history (time^2^ × maternal history, p = 0.036; Table 4). The interaction between time and BMI change (baseline BMI − BMI at 24 months) for memory did not reach significance (p = 0.127), although a three-way interaction between time, sex, and BMI change was significant at the quadratic level (p = 0.011; Table 4).

Executive trajectories (ADNI-EF) were significantly impacted by conversion (time × conversion, p < 0.001) and maternal history (time × maternal history, p = 0.026), with a trend-level interaction with age (time × age, p = 0.092) and a significant quadratic interaction with BMI change (time^2^ × BMI change, p = 0.038; Table 4). A time × conversion × BMI-change interaction was also significant for EF (p = 0.021; Table 4).

Supplementary Figures provide additional graphical representations of BMI, memory, and executive function trajectories across relevant subgroups, including stratification by maternal history, sex, age, and conversion status.

## Discussion

The findings of this study partially confirm our initial hypothesis and support the notion that maternal history (MH) of Alzheimer’s disease contributes to a distinct longitudinal clinical and anthropometric-metabolic profile in individuals with MCI. Consistent with previous literature, we observed that weight and BMI declined more markedly in participants who converted to AD^17,19^, and this decline was particularly pronounced over time in those with maternal history. The association between maternal history and BMI loss remained significant after adjusting for APOE4 status, age, sex, and conversion, suggesting that maternal lineage influences BMI-related trajectories independent of traditional genetic risk factors.

Weight loss has been widely described as an early, non-cognitive manifestation of AD and has been associated with subsequent progression to dementia^17,19^. Our results reinforce this concept by showing that greater BMI decline tended to co-occur with worse memory trajectories, although this association did not reach statistical significance in the main adjusted longitudinal models. Notably, sex-specific quadratic effects were observed, suggesting that the relationship between BMI decline and memory performance may be non-linear and biologically heterogeneous. These findings are compatible with the hypothesis that anthropometric-metabolic disruption and hippocampal vulnerability may share partially overlapping mechanisms, potentially involving mitochondrial dysfunction or impaired cerebral energy homeostasis—mechanisms previously proposed in the context of maternal transmission^2,3^.

Another key finding is the differential cognitive trajectory associated with maternal history. Participants with MH showed subtle but consistent longitudinal differences characterized by relatively greater involvement of memory-related performance, accompanied by relative preservation of executive function across follow-up. While baseline memory scores did not differ significantly by family history, maternal history was associated with a subtle non-linear pattern of memory change over follow-up, suggesting a distinct temporal trajectory rather than an early cross-sectional deficit. This profile is broadly consistent with prior neuroimaging studies reporting preferential temporomedial vulnerability in individuals with maternal history^4–7^ although direct anatomical inference cannot be made from the present data.

Conversely, participants without MH—particularly at older ages—exhibited more pronounced executive decline over time, a pattern that may reflect greater frontal or subcortical involvement, potentially influenced by vascular, anthropometric-metabolic, or lifestyle-related factors. Although these mechanisms were not directly assessed, the divergence in executive trajectories supports the concept of heterogeneous prodromal AD phenotypes.

Importantly, both APOE4 and maternal history independently contributed to BMI and cognitive trajectories, but their effects appeared to operate through partially distinct pathways. APOE4 was associated with steeper overall decline in BMI and cognitive performance, whereas maternal history was linked to more domain-specific and temporally non-linear effects, particularly affecting memory and BMI-related change. This distinction reinforces the idea that maternal transmission mechanisms extend beyond APOE4-related risk and may involve mitochondrial inheritance or epigenetic regulation^2,3^ although recent studies have reported mixed results regarding preferential maternal transmission^10,11^.

The multivariate longitudinal analyses further demonstrated that maternal history interacts with age and BMI change to influence cognitive trajectories, underscoring the complex, multidimensional nature of prodromal AD progression. The presence of quadratic interactions suggests that linear models may not fully capture these dynamics, and that family-history stratification may help to identify biologically meaningful subgroups within MCI.

This study has several limitations. First, as this was an exploratory analysis, no a priori sample size calculation was performed, and some family-history subgroup comparisons—particularly paternal and dual history— were likely underpowered to detect small effects. Family history was self-reported, which may introduce misclassification bias. Additionally, the absence of biomarker integration (amyloid, tau, or neurodegeneration imaging) limits direct attribution of the observed changes to specific AD pathophysiological processes. Although AD biomarkers were not available in the present analysis, the longitudinal clinical design, conversion status, and ADNI cognitive composites support the relevance of BMI decline as an accessible clinical marker of heterogeneity in MCI. Finally, BMI and body weight are coarse anthropometric measures that do not distinguish between lean mass, fat mass, or fluid changes, precluding conclusions about the specific biological substrates underlying the observed weight loss.

Overall, these findings suggest that maternal history is associated with a distinctive prodromal profile within MCI, characterized by accelerated BMI decline, differential memory trajectories, and relative preservation of executive function. Incorporating maternal history into clinical and research frameworks may improve phenotypic stratification in MCI and contribute to a more nuanced understanding of heterogeneous pathways in the early stages of Alzheimer’s disease.

## Supplementary Material

Supplementary Files

This is a list of supplementary files associated with this preprint. Click to download.

- Supplementarymaterial.pdf

## Figures and Tables

**Table 1:** Distribution of study variables according to conversion status from mild cognitive impairment to Alzheimer’s disease.

Variables		Stable MCI (n = 270)	Converted to AD (n = 127)	p-value	Total (n = 397)
Sex	Male	179 (66.3%)	77 (60.6%)	0.323	256 (65)
	Female	91 (33.7%)	50 (39.4%)		141 (35)
APOE4	Negative	142 (52.6%)	43 (33.9%)	0.001	185 (47)
	Positive	128 (47.4%)	84 (66.1%)		212 (53)
Maternal history	No	179 (78.5%)	81 (76.4%)	0.668	260 (78)
	Yes	49 (21.5%)	25 (23.6)		74 (22)
Age		78.7 ± 7.53	78.5 ± 7.35	0.830	78.6 ± 7.5
Age group	< 70	39 (14.4)	15 (11.8)	0.412	54 (14)
	70–80	113 (41.9)	62 (48.8)		175 (44)
	> 80	118 (43.7)	50 (39.4)		168 (42)
BMI	Baseline	26.2 ± 3.92	25.8 ± 4.10	0.283	26.1 ± 4.0
	12 months	26.2 ± 4.00	25.7 ± 3.87	0.285	26.0 ± 3.9
	24 months	26.2 ± 4.25	25.4 ± 3.71	0.114	25.9 ± 4.1
Weight	Baseline	76.8 ± 14.46	74.3 ± 13.77	0.105	76.0 ± 14.27
	12 months	76.5 ± 14.24	74.1 ± 13.90	0.124	75.7 ± 14.14
	24 months	76.8 ± 14.68	73.5 ± 13.49	0.055	75.5 ± 14.31
MEM	Baseline	0.055 ± 0.589	−0.389 ± 0.447	< 0.001	−0.09 ± 0.58
	12 months	0.081 ± 0.757	−0.569 ± 0.478	< 0.001	−0.15 ± 0.74
	24 months	0.093 ± 0.794	−0.821 ± 0.568	< 0.001	−0.25 ± 0.84
EF	Baseline	0.083 ± 0.785	−0.314 ± 0.711	< 0.001	−0.04 ± 0.78
	12 months	0.148 ± 0.889	−0.537 ± 0.809	< 0.001	−0.10 ± 0.92
	24 months	0.233 ± 0.911	−0.803 ± 0.916	< 0.001	−0.16 ± 1.04
MMSE	Baseline	27.23 ± 1.773	26.61 ± 1.714	< 0.001	27.03 ± 1.78
	12 months	27.37 ± 2.433	24.67 ± 2.809	< 0.001	26.40 ± 2.88
	24 months	27.12 ± 2.889	22.50 ± 3.808	< 0.001	25.38 ± 3.96

**Table 2:** Characteristics according to family history

		Family history					Total
		Maternal only (n = 74)	Maternal and paternal (n = 5)	Paternal only (n = 22)	None (n = 233)	p-value	
Sex	Male	43 (58%)	4 (80%)	13 (59%)	152 (65%)	0.572	212 (64%)
	Female	31 (42%)	1 (20%)	9 (41%)	81 (35%)		122 (36%)
APOE4	Negative	25 (34%)	-	8 (36%)	123 (53%)	0.003	156 (47%)
	Positive	49 (66%)	5 (100%)	14 (64%)	110 (47%)		178 (53%)
Age		76.8 ± 7.0	79.2 ± 9.1	75.5 ± 8.0	79.4 ± 7.1	0.009	78.6 ± 7.3
BMI	Baseline	25.6 ± 3.84	25.6 ± 3.27	25.9 ± 4.34	26.1 ± 3.87	0.877	26.0 ± 3.88
	12 months	25.3 ± 3.70	26.8 ± 3.51	25.6 ± 3.34	26.0 ± 3.94	0.662	25.8 ± 3.83
	24 months	25.2 ± 3.82	25.2 ± 3.78	26.0 ± 3.10	26.0 ± 4.08	0.605	25.8 ± 3.95
MEM	Baseline	−0.01 ± 0.58	0.17 ± 0.73	−0.17 ± 0.54	−0.09 ± 0.58	0.707	−0.10 ± 0.58
	12 months	−0.23 ± 0.70	−0.15 ± 0.96	−0.28 ± 0.78	−0.12 ± 0.70	0.618	−0.16 ± 0.71
	24 months	−0.27 ± 0.68	−0.53 ± 1.42	−0.35 ± 0.74	−0.28 ± 0.85	0.898	−0.29 ± 0.82
EF	Baseline	0.16 ± 0.73	−0.17 ± 1.38	0.00 ± 0.81	−0.09 ± 0.76	0.118	−0.03 ± 0.77
	12 months	0.15 ± 0.86	−0.25 ± 1.64	−0.04 ± 0.90	−0.18 ± 0.91	0.078	−0.10 ± 0.92
	24 months	0.16 ± 0.87	−0.70 ± 2.09	−0.09 ± 1.03	−0.26 ± 1.04	0.032	−0.15 ± 1.04
MMSE	Baseline	27.1 ± 1.84	27.2 ± 2.05	27.6 ± 1.26	27.0 ± 1.82	0.597	27.1 ± 1.79
	12 months	26.2 ± 2.90	25.4 ± 2.61	26.7 ± 2.72	26.5 ± 2.93	0.698	26.4 ± 2.90
	12 months	25.3 ± 3.31	23.4 ± 4.77	26.2 ± 3.32	25.4 ± 4.18	0.565	25.4 ± 3.94

**Table 3:** Repeated-measures ANOVA results for longitudinal BMI trajectories

		BMI		
Source	Effect	Mean square	F	p-value
Time	Linear	6.879	5.609	**.019**
	Quadratic	.993	1.917	.168
Time × Sex	Linear	1.709	1.394	.239
	Quadratic	2.017	3.896	**.050**
Time × APOE4	Linear	6.009	4.899	**.028**
	Quadratic	.048	.093	.761
Time × Conversion status	Linear	6.556	5.345	**.022**
	Quadratic	2.117	4.088	**.044**
Time × Family history	Linear	9.776	7.971	**.005**
	Quadratic	.006	.012	.913
Time × Age	Linear	5.365	4.374	**.038**
	Quadratic	.749	1.446	.231
Time × APOE4 × Conversion status	Linear	6.615	5.393	**.021**
	Quadratic	.031	.061	.806
Time × APOE4 × Age	Linear	5.196	4.236	**.041**
	Quadratic	.025	.047	.828
Time × Family history × Age	Linear	8.788	7.165	**.008**
	Quadratic	.009	.018	.894
Error(time)	Linear	1.226		
	Quadratic	.518		

**Table 4:** Repeated-measures ANOVA results for longitudinal memory (MEM) and executive function (EF) trajectories

		ADNI-MEM			ADNI-EF		
Source	Effect	Mean square	F	p-value	Mean square	F	p-value
Time	Linear	.087	.875	.351	.096	.773	.380
	Quadratic	.027	.586	.445	.016	.240	.624
Time × Sex	Linear	.324	3.242	**.073**	.364	2.940	**.088**
	Quadratic	.003	.066	.797	.179	2.683	.103
Time × APOE4	Linear	.317	3.171	**.076**	.400	3.228	**.074**
	Quadratic	.099	2.181	.141	.326	4.898	**.028**
Time × Conversion status	Linear	2.558	25.607	**< .001**	5.035	40.616	**< .001**
	Quadratic	.006	.133	.716	.031	.462	.497
Time × Family history	Linear	.010	.104	.747	.625	5.041	**.026**
	Quadratic	.201	4.434	**.036**	.009	.141	.708
Time × Age	Linear	.002	.020	.888	.011	.092	.762
	Quadratic	.016	.350	.555	.011	.160	.690
Time × BMI change	Linear	.234	2.346	.127	6.748E-5	.001	.981
	Quadratic	.016	.363	.547	.290	4.365	**.038**
Time × APOE4 × Conversion status	Linear	.017	.169	.681			
	Quadratic	.290	6.399	**.012**			
Time × Family history × Age	Linear				.130	1.052	.306
	Quadratic				.206	3.138	**.078**
Time × Sex × BMI change	Linear	.171	1.713	.192			
	Quadratic	.296	6.535	**.011**			
Time × Sex × Conversion status	Linear				.006	.046	.830
	Quadratic				.352	5.291	**.022**
Time × Conversion status × BMI change	Linear				.665	5.362	**.021**
	Quadratic				.018	.264	.608
Error(time)	Linear	.100			.124		
	Quadratic	0.45			.66		

## Data Availability

The datasets analyzed during the current study are available from the Alzheimer’s Disease Neuroimaging Initiative (ADNI) repository and can be accessed upon reasonable request at: https://adni.loni.usc.edu/
